# Insulin dysregulation plays a critical role in colon inflammation: a bioinformatics approach 

**Published:** 2018

**Authors:** Nosratollah Naderi, Mona Zamanian Azodi, Elahe Daskar Abkenar, Mohammad Shahidi Dadras, Ramin Talaei

**Affiliations:** 1 *Gastroenterology and Liver Diseases Research Center, Research Institute for Gastroenterology and Liver Diseases, Shahid Beheshti University of Medical Sciences, Tehran, Iran*; 2 *Student Research Committee, Proteomics Research Center, Shahid Beheshti University of Medical Sciences, Tehran, Iran*; 3 *Foodborne and Waterborne Diseases Research Center, Research Institute for Gastroenterology and Liver Diseases, Shahid Beheshti University of Medical Sciences, Tehran, Iran.*; 4 *Skin Research Center, Shahid Beheshti University of Medical Sciences, Tehran, Iran*; 5 *Modarres Hospital, Shahid Beheshti University of Medical Sciences, Tehran, Iran *

**Keywords:** Colorectal, Inflammation, Transcriptome, Protein interaction maps

## Abstract

**Aim:**

Evaluating and screening of genes related to colorectal inflammation of mice for finding critical ones in this disease was the aim of this study.

**Background:**

Many studies are shown direct relationship between inflammation and colorectal cancer onset and development. Several molecular aspects of inflammation are investigated to discover molecular mechanism of this disease.

**Methods:**

Profiles of differentially expressed genes (DEGs) of mice inflamed colorectal tissue in comparison with normal samples are obtained from Gene Expression Omnibus (GEO) database. The significant and characterized DEGs were screened via protein-protein interaction (PPI) network. Hubs of the network were determined and backbone network was constructed. Moreover, action network for the critical nodes was constructed and analyzed.

**Results:**

Eight central genes including IL6, ALB, PRDM10, AKT1, GAPDH, IL8, INS and TNF were determined as hub nodes. Findings indicate that insulin plays critical role in regulation of hub genes. This finding shows association between inflammation and metabolism dysregulation. Except PRDM10 and GAPDH, the other hubs show considerable regulatory effects on each other.

**Conclusion:**

Inflammation of colorectal tissue is strongly depended on metabolism especially to insulin function.

## Introduction

 Inflammation has a direct connection with development of cancer. In this respect, accompanied studies support these relationships from genetics, pharmacological, and epidemiological perspectives (1). On the other hand, colorectal cancer (CRC) as the fourth most frequent type of cancers in the U.S. and the most cause of cancer associated-death in the world accounts for 600,000 cases yearly (2, 3). There are some known genetics and environmental risk factors for colon cancer onset and development (4). For instance, some molecules such as microRNAs are introduced as early detection biomarkers for CRC (5). Inflammation as one of which, has an important role in colon tumor progression, invasion, and metastasis (2) via a network of inflammatory elements including cytokines, distinct immune cells, chemokines, and mediators of immune response (2). There are some evidence about association between metabolism disorders especially insulin dysregulation and inflammation (6). Based on this understanding, application of NSAIDs has been shown valuable in dealing with cancer. Inflammation could be triggered from different reasons (7) and with different shapes in colorectal tissue including ulcerative colitis and Crohn's disease as inflammatory bowel disease (IBD) (1, 8). The association between colitis and colon cancer is called UC-CRC. The risk of cancer from UC increases after 8-10 years at the diagnosis time of UC (9). The important thing is that the contribution of inflammation in this domain has been remained elusive. The study of molecular concept could be promising to decipher the mechanisms and understanding the place of the contributing molecular factors (4). Genomics and proteomics have identified some markers of interest including RNAs and microRNAs. Some of which are miR-21, miR-193a-3p, IRS, and mitochondrial regulator protein such as STEAP4 (3, 8). There is a need for more established and reliable biomarkers in this field. One of the novel approaches is the complementary study known as bioinformatics. This method could be applied for evaluating high throughput data and adding some more information for this category (10). The information could be beneficial for developing the knowledge of interacting biomarkers and pinpoint the most crucial ones. One of which is the network-based approach that identifies the potential biomarkers linked with the disease of study (11). In this way, genes are distinguished based on their central contribution in a network of interacting agents. 

Therefore, the aim of this study is to analysis the protein-protein interaction (PPI) network of inflammation in colon tissue to possibly get a better view of transition to tumorigenesis of colon. 

## Methods

**Data collection**

Microarray Data was obtained from GEO database; series GSE31106, GPL1261, GSM770092-94 as normal colorectal mucosa group and GSM770095-97 as inflamed colorectal mucosa group. Five-week-old male mice were intraperitoneal injected with 10mg/kg Azoxymethane and treated three cycles with Dextran sulfate sodium (2%, 1.5%, and 1.5%). Instead, the control group were injected with saline and treated with disttiled water drinking. After two weeks, colorectal tissue was collected and evaluated microscopically. The extracted RNAs were detected by Affymerix GeneChip Mouse Genome 430 2.0 Array. The 250 top significant DEGs were selected based on p-value less than 0.05 via GEO2R (https://www.ncbi.nlm.nih.gov/geo/geo2r/). 

The uncharacterized DEGs were excluded and the rest genes with the fold change (FC) less than 0.5 and more than 2 were identified as significant DEGs candidates then used as an interactome units. 

**PPI network analysis**

The selected DEGs were included in PPI network via STRING database and the network was constructed by Cytoscape software version 6.3.2. Network Analyzer plugin of Cytoscape was used to analysis the network (12-14). The nodes with higher value of degree (mean+2SD) were determined as hub nodes. Backbone network was created for hubs and analyzed to confirm hub-nodes (15). Action map network of hub nodes was made by CluePedia v 1.5.0 application of Cytoscape software (16). The action pattern was built via directed edges to map expression, binding, activation, and inactivation actions.

**Statistical analysis**

Value distribution of gene expression profiles of groups of samples are matched via boxplot analysis to show if they are normalized and cross-comparable for continuing the analysis. In addition, p-value <0.05 was considered for significant findings. 

## Results

**Figure 1:**
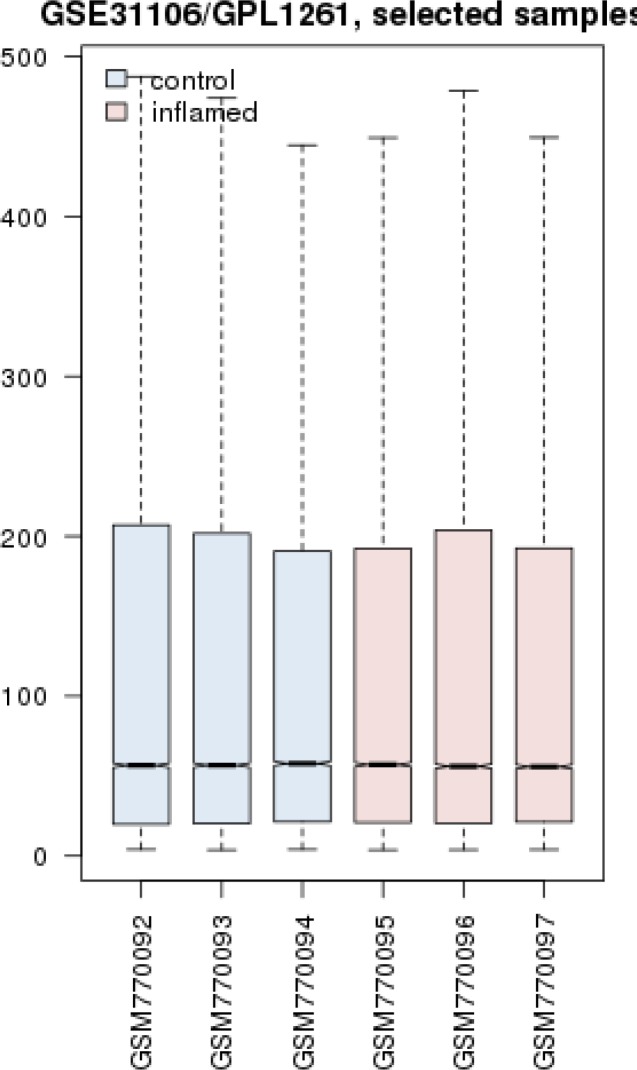
Box plot analysis of three treated rats relative to three health controls. Blue colored boxes represent control and pink ones represent inflamed samples. Lateral axis: names of samples, longitudinal axis: the genes

**Figure 2:**
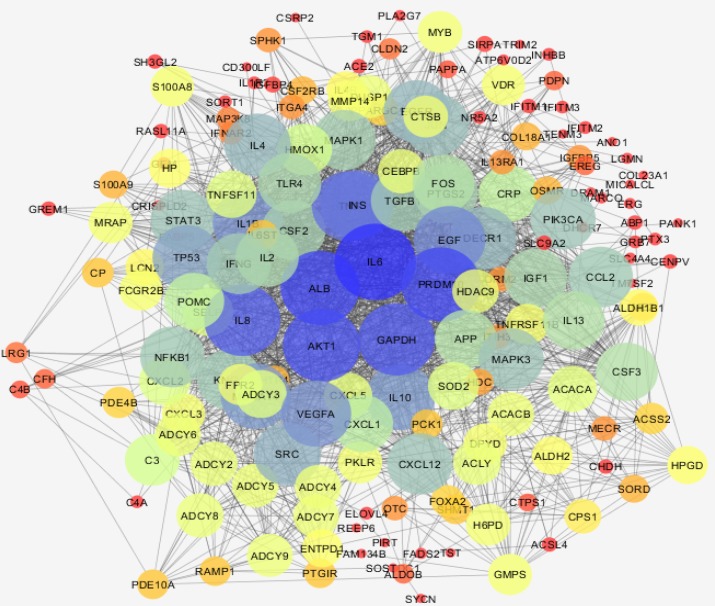
Main connected component of treated rats relative to controls network is presented. Bigger size and color from red to blue refer to higher degree values

Gene expression profiles of three inflamed samples were compared by three healthy normal rats via box plot analysis. As it is showed in the figure 1 the samples are median center and comparable. Among top 250 significant DEGs which differentiate treated rats from healthy controls, 9 genes were not characterized. 241 query genes were imported to STRING to construct PPI network. A number of 67 genes were not recognized by STRING and the 174 remained genes interacted in poor style so the network was not very informative. A number of 50 neighbor genes were added to 174 query genes and the network including 46 isolated nodes, 3 paired, and a main connected component counting 172 nodes were constructed. The main connected component is shown in the figure 2. The network was analyzed and eight hub nodes were identified (see table 1). To validate findings, a backbone network regarding the eight hub nodes by searching via general databases was constructed. 

**Table 1:** Hubs of Main connected component of treated rats relative to controls network are presented

R	Gene Name	Description	Degree
1	IL6	interleukin 6	81
2	ALB	Albumin	77
3	PRDM10	PR domain containing 10	77
4	AKT1	v-akt murine thymoma viral oncogene homolog 1	75
5	GAPDH	glyceraldehyde-3-phosphate dehydrogenase	74
6	IL8	interleukin 8	73
7	TNF	tumor necrosis factor	70
8	INS	Insulin	69

**Table 2:** Hubs of backbone network are presented

R	display name	description	Degree
1	INS	Insulin	100
2	AKT1	v-akt murine thymoma viral oncogene homolog 1	69
3	TNF	tumor necrosis factor	67
4	PRDM10	PR domain containing 10	65
5	ALB	Albumin	63
6	IL6	interleukin 6 (interferon, beta 2)	58
7	INSR	insulin receptor	57
8	GAPDH	glyceraldehyde-3-phosphate dehydrogenase	56

**Figure 3:**
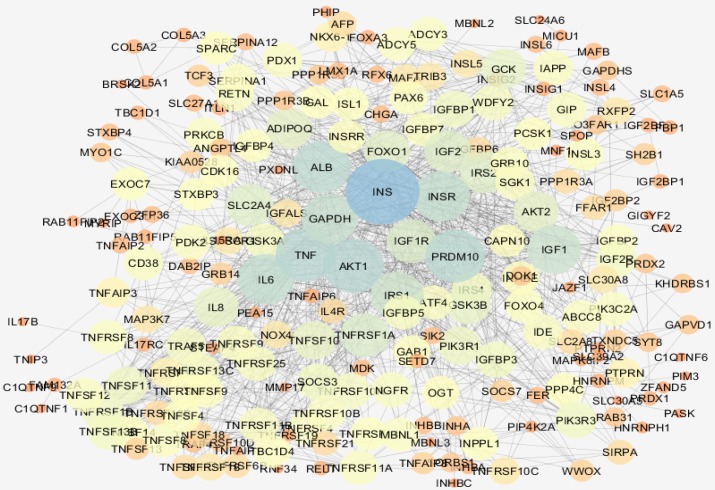
Backbone network of hub nodes of main connected component of inflamed samples relative to controls network is presented. Bigger size and color from red to blue refer to higher degree values

**Figure 4:**
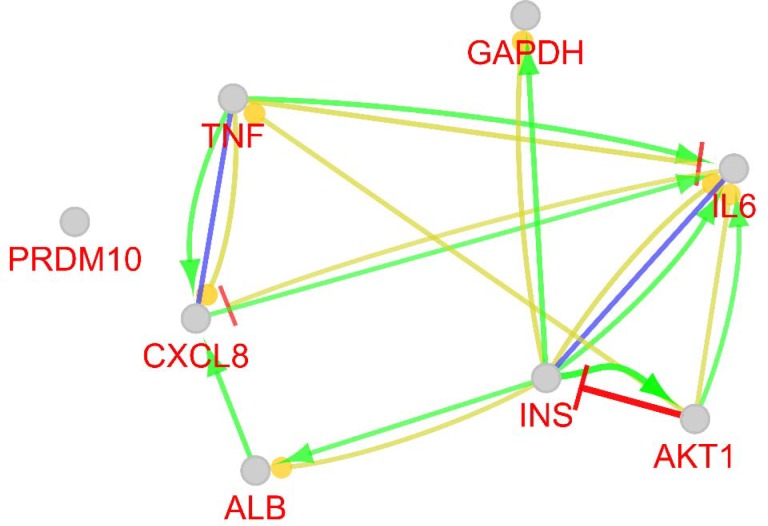
Action map of the eight hubs of main network including expression, binding, activation, and inhibition are shown in yellow, blue, green, and red, respectively

As it is illustrated in the figure 3, the network including a main connected component characterized with 218 nodes, one paired nodes, and 34 isolated ones were obtained. Hub nodes of backbone network are tabulated in the table 2. Action map of the eight hubs of main network (not backbone network) is shown in the figure 4. Expression, binding, activation, and inhibition properties of hubs relative to each other are schemed in this figure. As depicted in table 2, INS, AKT1, TNF, PRDM10, ALB, IL6, and GAPDH are the common central nodes between backbone network and the main connected component of network.

## Discussion

Inflammation is one of the important incidences in digestive system. In colon tissue, IBD is one of which that can result in a cancerous condition. Researchers are aimed to understand the mechanisms of inflammation and its development to tumorigenesis. Recently PPI network analysis is attracted attention of scientists (17, 18) as in this work, induce of inflammation in colorectal tissue of mice is compared to normal condition in this regard via analysis expression profiling of DEGs. As it is depicted in the figure 1, gene expression profiles of groups of samples are comparable via box plot analysis. The DEGs are interacted to construct a scale-free network (see figure 1). However, built of network was dependent to 50 additional relevant genes to the query ones. Network analysis led to determine 8 central nodes; however, the hubs were not included among the query genes. IL6, ALB, PRDM10, AKT1, GAPDH, IL8, INS and TNF are the eight hub nodes that are tabulated in the table 1. To validate the 8 hubs for centrality properties, a backbone network was constructed (see figure 3). As it is shown in table 2, the hub nodes of backbone network are as INS, AKT1, TNF, PRDM10, ALB, IL6, INSR, and GAPDH. Comparison between hubs of the main and the backbone networks, indicated that 7 hubs of main network are involved in the backbone network as hub nodes. 

On the other hand, except IL8, all of the other hubs of the network are appeared in the backbone network as hubs. INSR, which is an insulin receptor, is apparently as hub node in the backbone network; however, it is well known that INSR is correlated to the INS. The construction of backbone network confirmed the introduced hub nodes are the central genes that are involved in onset and development of colorectal tissue inflammation. For getting more resolution of hub nodes, action analysis of them were analyzed. 

The regulatory effect of hub nodes on each other is illustrated in figure 3 and highlighted considering expression, binding, activation and inhibition actions. At first glance, inhomogeneous behavior of genes on each other is obvious. For instance, GAPDH is regulated by INS for expression and activation actions while it has no regulatory effect on the other genes. Thus, GAPDH cannot be considered as a regulatory element in the network of inflamed colorectal tissue. PRDM10, the other hub node, that is the third important central node in table 1, not only did not show any regulatory effect on the elements of hub nodes, but also it is not regulated by any of agents of this action network. Therefore, this gene can be considered as an isolated gene in this action network. IL6 and IL8 (CXCL8), are two chemokines that except mutual regulatory effect on each other, have no regulatory impact on the other hub nodes. These two genes are affected by the numbers of the central nodes. INS and TNF, the highly influential genes, show regulatory effects on the central nodes. It can be concluded that the recent two genes are more prominent than the other nodes. INS is a key gene in metabolism pathways especially in regulation of glucose and lipids. The well-known disease related to the dysregulation of insulin is diabetes. It has been reported that insulin has some associations with colorectal cancers pathogenicity (19). Insulin is an interesting agent that is involved in different kinds of diseases. Some studies claim that insulin is correlated to the neurological disorders in including Alzheimer's disease and bipolar disorder (20, 21). In view of the fact that different diseases are correlated with metabolism dysfunction, it may be concluded that insulin has vast roles in many diseases (22). TNF, on the other hand, is known as a tumor necrosis factor that is usually related to the cancerous tissues. In a study that was aimed to reduce the colon inflammation, it was resulted in lessening of TNF expression (23). Two chemokines of IL6 and IL8 are main agents of immune and inflammatory system (24, 25). Yet, their presence in this suggested panel of 8 important genes is justified. The contribution of ALB and GAPDH, the two important housekeeping genes (26) refer to the overall changes in body maintenance due to the alteration of this condition based on inflammation occurrence (27). The close relationship between the recognized central panel of genes and inflammation indicates that regulation of these dysregulated genes possibly has key influence on inhibition of inflammation (28, 29). The oncogenesis features of some of these genes, shows that a patient with inflamed colorectal is susceptible to colon cancer onset. 

In conclusion, insulin accompanied with 7 genes of central properties, are responsible for integrity of inflamed colorectal tissue. Three features including metabolic, oncogenic and immunologic of inflamed colon tissue emphasized by this investigation.
